# The German Guidelines for the treatment of anxiety disorders: first revision

**DOI:** 10.1007/s00406-021-01324-1

**Published:** 2021-10-05

**Authors:** Borwin Bandelow, Antonia M. Werner, Ina Kopp, Sebastian Rudolf, Jörg Wiltink, Manfred E. Beutel

**Affiliations:** 1 Department of Psychiatry and Psychotherapy, University Medical Center Göttingen, University of Göttingen, von-Siebold-Str. 5, 37075 Göttingen, Germany; 2 Department of Psychosomatic Medicine and Psychotherapy, University Medical Center of the Johannes Gutenberg University Mainz, Mainz, Germany; 3 AWMF Institute for Medical Science Management, Mainz, Germany; 4 Helios Clinic for Psychiatry and Psychosomatic Medicine, Schleswig, Germany

**Keywords:** Guideline, Anxiety disorders, Panic disorder, Generalized anxiety disorder, Social phobia, Treatment, Psychotherapy, Drug treatment

## Abstract

Starting in 2019, the 2014 German Guidelines for Anxiety Disorders (Bandelow et al. Eur Arch Psychiatry Clin Neurosci 265:363–373, 2015) have been revised by a consensus group consisting of 35 experts representing the 29 leading German specialist societies and patient self-help organizations. While the first version of the guideline was based on 403 randomized controlled studies (RCTs), 92 additional RCTs have been included in this revision. According to the consensus committee, anxiety disorders should be treated with psychotherapy, pharmacological drugs, or their combination. Cognitive behavioral therapy (CBT) was regarded as the psychological treatment with the highest level of evidence. Psychodynamic therapy (PDT) was recommended when CBT was not effective or unavailable or when PDT was preferred by the patient informed about more effective alternatives. Selective serotonin reuptake inhibitors (SSRIs) and serotonin-noradrenaline reuptake inhibitors (SNRIs) are recommended as first-line drugs for anxiety disorders. Medications should be continued for 6–12 months after remission. When either medications or psychotherapy were not effective, treatment should be switched to the other approach or to their combination. For patients non-responsive to standard treatments, a number of alternative strategies have been suggested. An individual treatment plan should consider efficacy, side effects, costs and the preference of the patient. Changes in the revision include recommendations regarding virtual reality exposure therapy, Internet interventions and systemic therapy. The recommendations are not only applicable for Germany but may also be helpful for developing treatment plans in all other countries.

## Introduction

Anxiety disorders (Table 1) are the most prevalent psychiatric disorders and are associated with a high burden of illness [8]. Women are twice as frequently affected as men. With a 12-month prevalence of 10.3%, specific phobias are the most common anxiety disorders [18], however, individuals suffering from isolated phobias rarely seek treatment. The second most common type is panic disorder with or without agoraphobia (PDA; 6.0%), followed by social anxiety disorder (SAD) (2.7%) and generalized anxiety disorder (GAD; 2.2%). There is no evidence that anxiety disorders have occurred more frequently in recent years or decades [21, 22]. These disorders often co-occur with other anxiety disorders, depression, somatoform, personality, and substance abuse disorders [19].

**Table 1:** Anxiety disorders: short description according to ICD-10 classification [32]

Anxiety disorder	Description
Panic disorder F41.0	Anxiety attacks of sudden onset, with physical manifestations of anxiety (e.g., palpitations, sweating, tremor, dry mouth, dyspnea, feeling of choking; chest pain; abdominal discomfort; feelings; feeling of unreality, paresthesia, etc.) Panic attacks can arise out of the blue; however, many patients start to avoid situations in which they fear that panic attacks might occur
Agoraphobia F40.0 without panic disorder F40.00 with panic disorder F40.01	Fear of places where it might be difficult or embarrassing to escape if a panic attack should occur (crowds, on public transport, or in closed spaces, e.g., elevators). Fear of being alone is also common. The presence of a companion may reduce anxiety
Generalized anxiety disorder F41.1	Patients suffer from somatic anxiety symptoms (tremor, palpitations, dizziness, nausea, muscle tension, etc.) as well as from difficulty concentrating, nervousness, insomnia, and other psychic symptoms. Constant worry, e.g., that they (or a relative) might have an accident or become ill
Social phobia F40.1	These patients are afraid of situations in which they are the center of attention—e.g., public speaking, visits to authorities, conversations with superiors on the job, or with persons of the opposite sex. They are afraid of appearing clumsy, embarrassing themselves, or being judged negatively
Specific (isolated) phobias F40.2	Phobias which restricted to circumscribed situations, often related to animals (e.g., cats or spiders), or other natural phenomena (e.g., blood, heights, deep water)
Mixed anxiety and depressive disorder F41.2	The simultaneous presence of anxiety and depression, with neither predominating. However, neither component is sufficiently severe to justify a diagnosis of anxiety or depression in itself. If the diagnostic criteria for anxiety or depression (or both) are fulfilled, then the corresponding diagnosis should be made, rather than mixed anxiety and depressive disorder

Current conceptualizations of anxiety disorders posit an interaction of specific genetic vulnerabilities which manifest in neurobiological alterations and environmental factors (including childhood adversity, stress, or trauma). An abundance of high-quality research addressed the neurobiological causes of anxiety disorders, including research in neuroimaging, genetics, neurochemistry (neurotransmitters such as serotonin, norepinephrine, dopamine or GABA, neuropeptides such as cholecystokinin, neurokinins, atrial natriuretic peptide, or oxytocin, the HPA axis, neurotrophic factors such as NGF and BDNF, immunology, CO_2_ hypersensitivity, neurophysiology and neurocognition) [5, 6]; however, at present, none of the putative biomarkers has proved sufficient and specific as a diagnostic tool for anxiety disorders.

A consensus panel of German experts started to develop the “S3 guideline” for anxiety disorders in the year 2008. The term S3 refers to the highest quality requirements as defined by systematic evidence search and a consensus statement [2]. The present paper introduces the first revision of the German Guidelines for Anxiety Disorders [7]. Since 2014, many new randomized controlled studies (RCTs) for the treatment of anxiety disorders have been published. Recommendations on newly introduced treatment strategies were also examined, including Internet interventions and virtual reality exposure therapy. The guideline revision was published in 2021 and is available online [4].

In Germany, costs of the treatment of anxiety disorders are reimbursed by the statutory health insurance providers. Drug treatment is fully reimbursed, and so are defined contingents of psychotherapy sessions. Psychotherapy is provided by psychotherapists, including certified physicians or psychologists.

Anxiety disorders are mostly treated on an outpatient basis. Indications for hospitalization include suicidality, chronic anxiety disorders unresponsive to standard outpatient treatments, or marked comorbidity, e.g., with major depression, personality disorders or substance abuse.

## Methods

The guideline expert panel consisted of 35 specialists for psychiatry and psychosomatics/psychotherapy, general practitioners, psychologists, and other members from all relevant professional societies and patient self-help organizations (*n* = 29) involved in the treatment of anxiety disorders in Germany (for a list, see [4]).

Pre-existing international guidelines on the treatment of anxiety disorders were searched using the Guidelines International Network [15]. Since the first version, new widely recognized international guidelines have appeared or have been revised [1, 3, 20, 23, 26]. Recommendations in these guidelines were screened but the expert panel also performed its own research when discrepancies between existing guidelines were found, when certain subject areas were not adequately covered, or when new trials potentially altering the evidence level of a treatment approach were published since the publication of the reference guidelines. For the revision, randomized controlled studies (RCTs) on the treatment of anxiety disorders were searched which were published between 16/09/2013 and 20/06/2019. Literature search methodology followed the principles of the PRISMA Statement [24]. Inclusion criteria for RCTs were: original publication in a peer-reviewed journal; RCTs on the treatment of anxiety disorders in adults defined according to ICD or DSM (panic disorder/agoraphobia, generalized anxiety disorder, social phobia, or specific phobia); not exclusively subgroup analysis; use of a control group (i.e. for drug trials, a placebo or a validated reference drug; for psychotherapy trials, a waiting list, an active control or ‘psychological’ placebo, i.e., a supportive conversation with the patient, without use of specific psychotherapeutic techniques; treatment as usual (TAU) or another established form of psychotherapy as reference. Only commercially available drugs approved by the EMA (European Medicines Agency) for an anxiety indication were included in the recommendations.

**Fig. 1:**
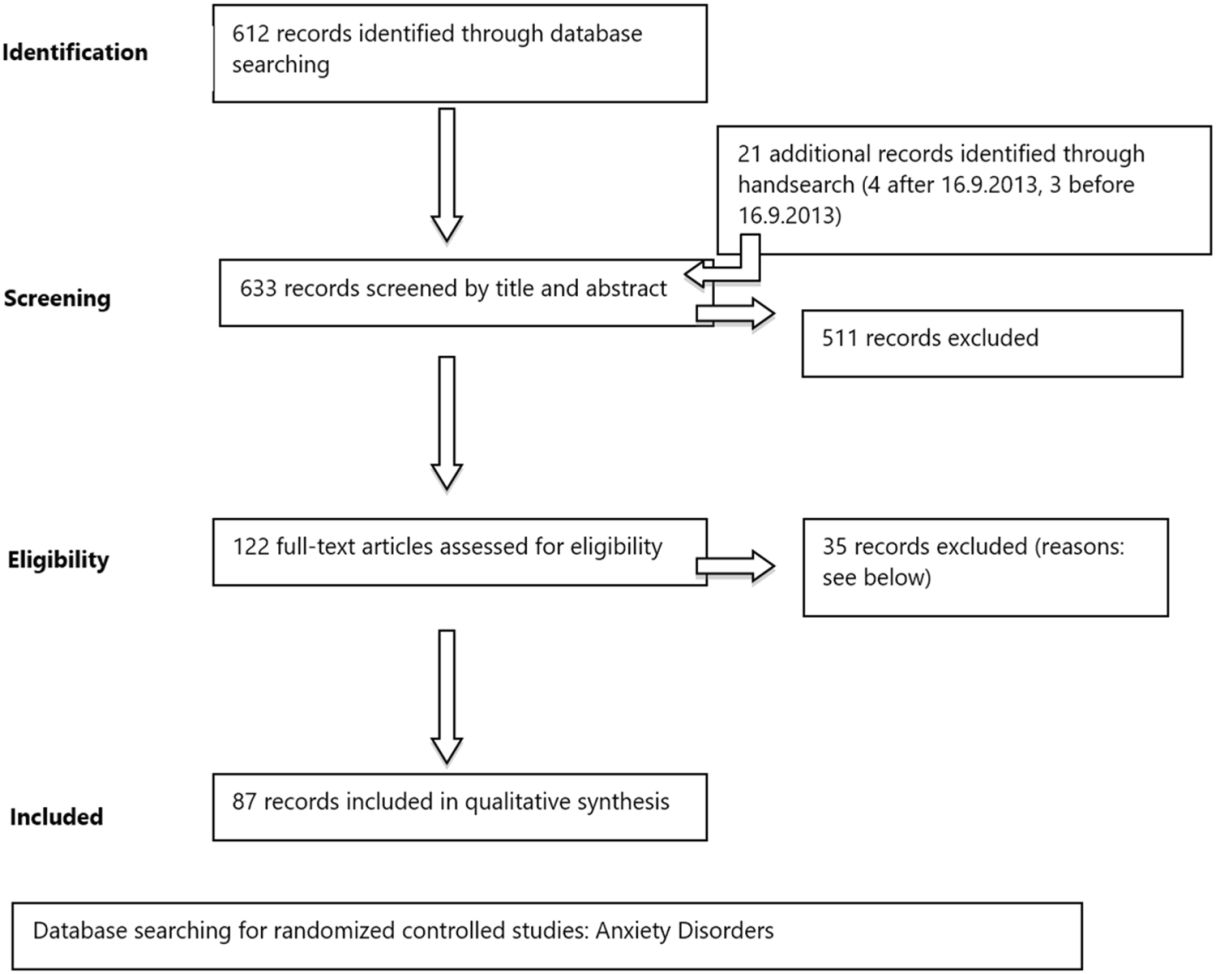
PRISMA statement [24]. Newly included studies since 16/09/2013

In the original version, a total of 1299 records were screened by titles and abstracts. For the revised version, 638 newly found records identified through database and hand search were screened by title and abstract (see PRISMA statement; Fig. 1). Records were eliminated for the following reasons: only meeting abstract, letter, review, meta-analysis, pooled study, double publication, secondary analyses, drug or herbal preparation not licensed, no DSM/ICD anxiety disorder diagnosis, patients with mixed anxiety disorders, only comorbid patients, treatment of subgroups (e.g., only students; exception: studies with patients ≥ 65 years were analysed separately), children/adolescents < 18; treatment of non-responders/adjunctive treatment of non-responders, augmentation, or drug combination treatments, sample size of any of the arms in a study of < 10 at baseline. After reading full texts, additional articles were eliminated because of qualitative shortcomings. The quality of the RCTs was evaluated following the SIGN Statement criteria [30], including blinding of investigators, equal conditions for treatment and control groups, use of standard assessment instruments, intent-to-treat analysis, adequate statistical power and correctness of statistical evaluation. Methodological flaws led to the exclusion of trials or to downgrading of their evidence level. Common reasons for downgrading the evidence grade included small sample size (particularly in non-inferiority comparisons) and inappropriate statistical methods. If a primary efficacy measure was not stated, results were re-evaluated using a Bonferroni-Holm correction [16] for multiple testing. Eventually, 92 new records fulfilling the inclusion criteria were included in the analysis.

The guideline protocol also allowed including results from open studies, case series, and single case reports whenever there were no sufficient RCTs available for certain decisions. However, there was no actual case in which a decision about an evidence level had to be based on such studies. While the evidence categories were based exclusively on the efficacy of the various treatments studied, the recommendation grades also took risks into account, e.g., adverse effects of drugs (Table 2).

**Table 2:** Evidence levels and recommendations grades

Level of evidence	Definition
Ia	Evidence from a meta-analysis of at least three randomized controlled trials (RCTs)
Ib	Evidence from at least one RCT or a meta-analysis of fewer than three RCTs
IIa	Evidence from at least one methodologically sound, non-randomized controlled trial
IIb	Evidence from at least one methodologically sound, quasi-experimental descriptive study
III	Evidence from methodologically sound, non-experimental observational studies, e.g., comparative studies, correlation studies, and case–control studies
IV	Expert committee reports or expert opinion and/or clinical experience of recognized authorities

Positive recommendation		Negative recommendation	
Recommendation grades			
A +	“Shall” recommendation: at least one RCT of good overall quality and consistency supports the recommendation directly, without extrapolation (evidence levels Ia and Ib)	A −	“Must not”: recommendation against the measure in question based on level Ia and Ib evidence
B +	“Should” recommendation: well-conducted clinical trials, other than RCTs, support the recommendation either directly (evidence levels II or III) or by extrapolation (evidence level I) if the studies lack direct connection to the specific topic	B −	“Should not”: recommendation against the measure in question based on level II and III evidence
0 +	“May” recommendation: expert committee reports or expert opinion and/or clinical experience of recognized authorities (evidence level IV) or extrapolation from evidence of levels IIa, IIb, or III. This recommendation grade indicates that no directly applicable clinical studies of sufficiently high quality are available for consideration	0 −	Data are lacking for the recommendation of the intervention
CCP +	Expert consensus/clinical consensus point: if no unequivocal evaluation of a relevant clinical topic was possible, recommendations were formulated by expert consensus	CCP −	Expert consensus/clinical consensus point: if no unequivocal evaluation of a relevant clinical topic was possible, recommendations were formulated by expert consensus point

According to the principles of the guideline, the level of evidence has been determined on the basis of available original studies. Only in the case of contradictory results, meta-analyses have been used.

All recommendations were agreed by voting of the group members. All participating societies had one vote, and all statements issued by the committee were only accepted if they received at least 75% of all votes cast. Moderation of the expert consensus meetings was done by the Association of Scientific Medical Societies (Arbeitsgemeinschaft der wissenschaftlichen medizinischen Fachgesellschaften, AWMF, www.awmf.org), a German overhead organization managing official guidelines for all fields of medicine.

Because of the large number of study reports included in the guideline (*n* = 606), references are not provided for every statement in this article; rather, the reader is referred to the full guideline text [4], in German only).

## Results

### Diagnosis

For the diagnosis of anxiety disorders diagnosed using ICD or DSM criteria should be used. For Germany, the International Classification of Diseases [32] in its German modification (ICD-10 GM) is the official diagnostic system. In primary care, anxiety disorders often go unrecognized [33]. Before diagnosing an anxiety disorder, other mental disorders, such as other anxiety disorders, major depression, personality and somatoform disorders, as well as physical illnesses such as coronary heart disease, pulmonary disease, and others have to be excluded.

### Treatment

Treatment is indicated when a patient fulfills criteria for an anxiety disorder as defined by ICD or DSM, shows marked distress or suffers from the sequelae resulting from the disorder (e.g., suicidality, secondary depression or substance abuse).

Patients should be educated about their diagnosis, the possible backgrounds and the principles of action of the available treatment approaches.

Anxiety disorders can be treated with psychotherapy, drug treatment and other interventions (Table 3). It is mandatory by law for treating psychologists and physicians to inform patients about the risks and benefits of available treatments and possible alternatives which are more effective or better tolerated. The treatment plan should be chosen after careful consideration of individual factors, e.g., the patient’s preference, previous successful or unsuccessful treatment attempts, illness severity, comorbidities including substance abuse or suicide risk, availability of treatment methods, costs, waiting periods and others. All interventions should be performed on the basis of an empathic and reliable therapeutic relationship. It is suggested that treatment success should be monitored with standard ratings scales.

**Table 3:** German Guideline for the treatment of anxiety disorders: treatment recommendations for anxiety disorders in adults

Treatment	Recommendation	Level of evidence	Recommendation grade
Psychotherapy and psychotropic drugs	Patients with PDA, GAD, or SAD should be offered: –Psychotherapy –Medication The preference of the well-informed patient should be respected. The patient should be informed, in particular, about the onset and duration of action, side effects, and availability of the different treatment approaches	Ia	A +
	If psychotherapy or psychotropic drugs were not effective, the other approach or a combination of both should be offered	Expert consensus	CCP +
Psychotherapy and other non-pharmacological options			
Cognitive behavioral therapy (CBT)	Patients with PDA, GAD, SAD, or specific phobias should be offered CBT	Ia	A +
Psychodynamic psychotherapy (PDT)	Patients with PDA, GAD, or SAD should be offered psychodynamic psychotherapy if CBT is unavailable or ineffective, or if they express a preference for psychodynamic psychotherapy after being informed about all available types of treatment	IIa	B +
Virtual reality exposure therapy	Patients with specific phobias (fear of spiders, heights, or flights) can be offered as an adjunctive measure to other standard treatments	Ib	CCP +
	Patients with social phobia can be offered as an adjunctive measure to other standard treatments	Expert consensus	CCP +
Systemic therapy	Patients with SAD can be offered systemic therapy if CBT or psychodynamic is unavailable or ineffective, or if they express a preference for systemic therapy after being informed about all available types of treatment	Expert consensus	0 +
Internet-based psychological interventions	Patients with PDA, GAD, or SAD should be offered Internet-based psychotherapeutic interventions (based on CBT for PDA, GAD, or SAD; based on psychodynamic therapy for SAD only) as an adjunctive measure to other standard treatments or to bridge the time until standard psychotherapy begins in the sense of a self-help strategy	Expert consensus	CCP +
Exercise (endurance training, e.g., running 5 km three times a week)	Patients with PDA can be given a recommendation for exercise (endurance training) as an adjunctive measure to other standard treatments	Expert consensus	CCP +
Patient self-help and family support groups	Patients and their families should be informed about self-help and family support groups and encouraged to participate, if appropriate	Expert consensus	CCP +

Medications	Drug	Anxiety disorder			Daily dose (mg)		
		PDA	GAD	SAD			
	Citalopram^1^	x			20–40	Ia	A +
	Escitalopram^2^	x	x	x	10–20	Ia	A +
	Paroxetine	x	x	x	20–50	Ia	A +
	Sertraline	x		x	50–150	Ia	A +
	Duloxetine		x		60–120	Ia	A +
	Venlafaxine	x	x	x	75–225	Ia	A +
Tricyclic antidepressant	Clomipramine (if drugs with a grade A recommendation are ineffective or poorly tolerated)	x			75–250	Ia	B +
Calcium modulator	Pregabalin		x		150–600	Ia	B +
Tricyclic anxiolytic	Opipramol (if drugs with a grade A or B recommendation are ineffective or poorly tolerated)		x		50–300	Ib	0 +
Azapirone	Buspirone (if drugs with a grade A or B recommendation are ineffective or poorly tolerated)		x		15–60	Ib	0 +
RIMA	Moclobemide (if drugs with a grade A or B recommendation are ineffective or poorly tolerated)			x	300–600	Expert consensus	CCP +

*PDA* panic disorder/agoraphobia, *GAD* generalized anxiety disorder, *SAD* social phobia, *CCP* clinical consensus point, *RIMA* reversible monoamine oxidase A inhibitor

^1^Do not exceed recommended dose (possible QT_C_ interval prolongation). Maximal dose with diminished hepatic function 30 mg/day, for older patients 20 mg/day

^2^Do not exceed recommended dose (possible QT_C_ interval prolongation). Maximal dose for persons over age 65, 10 mg/day

#### Psychotherapy

Of all psychological interventions, cognitive behavioral therapy (CBT) has by far the best body of evidence. In the case of phobic disorders, confronting the patients with their feared situations in exposure sessions is a crucial ingredient of the therapy. Group CBT has also been studied in RCTs, but there is not sufficient evidence to conclude that it is as effective as individual treatment. For patients with SAD, however, it seems reasonable to conduct self-assurance training in groups; thus, psychotherapy for SAD should include both personal and group therapy sessions.

For specific phobias, only studies with behavioural therapy exist, which should be performed as exposure treatment.

In comparison to CBT, the evidence for the efficacy of psychodynamic therapy (PDT) is weaker. RCTs on PDT were markedly fewer in number, and lower in quality, than those on CBT, and some comparison studies indicated superiority of CBT over PDT. According to the guideline, patients with PDA, GAD, or SAD should be offered PDT only if CBT was shown to be ineffective or is unavailable, or if the adequately informed patient expresses a preference for PDT.

Systemic therapy has recently become eligible for reimbursement by the German health care system. However, the few available studies had serious methodological flaws and inconsistent efficacy results; therefore, this treatment modality only received the “0” recommendation, indicating that it only should only be offered if standard treatments have failed or are not available.

Most efficacy studies used treatment manuals guiding the intervention strategy. Therefore, to maintain quality standards, it is recommended that psychotherapy in daily routine practice should also be manualized.

The guideline committee did not provide recommendations regarding the necessary duration or number of psychotherapy sessions due to the lack of reliable data. Published randomized studies on psychological therapies had an average study duration of 12.4 weeks [9], therefore, little is known about the additional benefits of psychotherapies that have 25, 50 or more sessions. An analysis performed by the guideline committee revealed that there is not sufficient evidence that longer therapies are more effective than shorter ones (for details, see original guideline).

Specific phobias can be treated in only a few exposure sessions. Most studies for the treatment of specific phobias (including fear of spiders, heights, or flying) had only one session lasting between 1 and 3 h, demonstrating that such short interventions are effective.

#### Internet-based psychological interventions (IPI)

In the recent years, numerous studies have investigated internet-based psychological interventions (IPI), most of which were based on CBT approaches. These usually involve no personal contact or only minimal E-mail or telephone contact with the study staff.

In most RCTs, IPIs were more effective than a waitlist control. However, evidence showing that IPIs are as effective as individual CBT with face-to-face contact is insufficient. Moreover, the efficacy results of IPI studies are mostly based on non-blinded self-ratings, which may lead to an overestimation of effects sizes due to expectation effects [11]. Therefore, the committee decided that IPIs should not be used as monotherapy but can be used to bridge a waiting period until face-to-face psychotherapy is available, or as an add-on self-help measure accompanying standard psychotherapy or medication treatment.

### Virtual and augmented reality exposure treatment

Virtual reality (VR) and augmented reality (AR) technologies have been introduced in the treatment of phobias. In AR exposure therapy, virtual elements are merged into the view of the physical world. Thus, the experience is more authentic, and costs are lower, because it is not necessary to program the complete virtual environment.

For PDA, there are not enough studies to support the use of VR. However, for SAD, VR exposure therapy can be used as an add-on self-help measure. For specific phobias (fear of spiders, heights, or flying), VR exposure therapy can be used when *in vivo* exposure is not available.

#### Pharmacotherapy

A large database of RCTs on the efficacy of medications for PDA, GAD, and SAD is available. Only for specific phobias, drug studies are scarce, and behavioral treatments should be preferred.

First-line drugs for anxiety disorders include the selective serotonin reuptake inhibitors (SSRI) and the serotonin-norepinephrine reuptake inhibitors (SNRI) (Table 3). Drug side effects are listed in Table 4. For panic disorder, the tricyclic antidepressant clomipramine may be a second-line option. The drug is as effective as the SSRIs and SNRIs but has more adverse effects. For GAD, the calcium modulator pregabalin was shown to be effective, but there are concerns about cases of overdoses and withdrawal syndromes associated with the drug. Therefore, pregabalin should not be used as the first option and should not be given to patients with a history of substance abuse.

**Table 4:** Adverse effects of anti-anxiety drugs

Medication class	Side effects
Selective serotonin reuptake inhibitors (SSRIs)	Jitteriness, nausea, restlessness, headache, fatigue, increased or decreased appetite, weight gain, weight loss, tremor, sweating, QT_C_ prolongation, sexual dysfunction, diarrhoea, constipation, and other side effects
Selective serotonin-noradrenaline reuptake inhibitor (SNRI)	Jitteriness, nausea, restlessness, headache, fatigue, increased or decreased appetite, weight gain, weight loss, tremor, sweating, sexual dysfunction, diarrhoea, constipation, urination problems, and other side effects
Tricyclic antidepressants (TCA)	Anticholinergic effects, somnolence, dizziness, cardiovascular side effects, weight gain, nausea, headache, sexual dysfunction, and other side effects
Pregabalin	Dizziness, somnolence, dry mouth, oedema, blurred vision, weight gain, constipation, euphoric mood, balance disorder, increased appetite, difficulty with concentration/attention, withdrawal symptoms after abrupt discontinuation, and other side effects
Buspirone	Dizziness, nausea, headache, nervousness, light-headedness, excitement, insomnia, and other side effects
Moclobemide	Restlessness, insomnia, dry mouth, headache, dizziness, gastrointestinal symptoms, nausea, and other side effects
Opipramol	Somnolence, dry mouth, tachycardia, dizziness, nausea, cardiac arrhythmias and other side effects

Only most common and relevant side effects included. For details, see the current summary of product characteristics

The guideline group advised against the use of benzodiazepines because of their abuse potential, despite their high effectiveness in treating anxiety. However, in exceptional cases (e.g., severe cardiac disease, suicidality, contraindications for standard medications, and other conditions), benzodiazepines can be used for a limited time period after their risks and benefits have been weighed carefully.

Other treatment approaches which were not considered as first- or second-line drugs due to lack of consistent evidence from RCTs, include the tricyclic opipramol (a drug which is only licensed in Germany and a few other countries), buspirone for GAD, and moclobemide for SAD.

Only in rare cases, emergency drug treatment is an option in treating acute panic attacks. Mostly, talking calmly with the patient and explaining that the attack is not due to a serious medical condition is sufficient. Only in severe cases, lorazepam 1.0–2.5 mg melting tablets may be given p.r.n.

Patients have to be informed about possible adverse effects, interactions, contraindications, and warnings, following the current summary of product characteristic.

Patients starting treatment with antidepressants (SSRIs, SNRIs, and TCAs) should be advised that these drugs generally take effect after a latency period of about 2 weeks (range 1–6 weeks). During the first weeks of treatment, some patients have a tendency to discontinue treatment with SSRIs and SNRIs due to initial jitteriness and nervousness. Compliance can be improved by informing patients about these potential adverse effects and by starting at half of the usually recommended dose. The drugs should be given in the morning or at midday to avoid insomnia which may occur during the first treatment weeks. In patients with hepatic impairment, a dosage adjustment or use of medications with primarily renal clearance (e.g., pregabalin) may be required.

The recommended medications have been studied in placebo-controlled relapse prevention studies mostly lasting from 6 to 12 month, as this is a requirement by the EMA for new drug application. According to these studies, it is advisable to continue drug treatment for 6 to 12 months after remission has occurred. In patients with a long history of recurrent and/or severe anxiety symptoms, longer treatments may be necessary.

To avoid discontinuation syndromes, the dose should be slowly tapered at treatment termination.

In patients who are unresponsive to medications, the addition of psychotherapy is generally recommended. If there is no response to the first drug after 4–6 weeks of treatment, a second standard drug should be given instead. In case of a partial response after 4–6 weeks, raising the dose can be considered first. Table 5 contains a stepwise plan for treatment options in case of drug inefficacy or intolerance. When medications are offered off-label to patients with treatment-unresponsive anxiety disorders which were effective in RCTs but were not licensed for the specific disorder (e.g. quetiapine or agomelatine), medicolegal issues have to be considered.

**Table 5:** Stepwise plan for drug treatment if the initial standard drug treatment was not effective or poorly tolerated (modified from [12])

Switch from one standard drug to another	Switch from one SSRI to another Switch from an SSRI to an SNRI, or vice versa Switch to a TCA Switch to pregabalin (only in GAD) or combination of SSRIs/SNRIs plus pregabalin	
Switch to non-standard drugs		
Switch to a drug that is approved for other anxiety disorders	Switch to pregabalin Switch to moclobemide, opipramol, or hydroxyzine Switch to a benzodiazepine (only in rare cases, when clinically justified)	
Switch to a drug (or drug combination) that is not approved for the anxiety disorder in question but has been found effective in RCTs	Panic disorder	Mirtazapine, quetiapine, phenelzine
	GAD	Quetiapine In refractory cases, addition of risperidone or olanzapine to treatment with an antidepressant
	Social phobia	Mirtazapine, gabapentin, pregabalin, olanzapine
Switch to a drug (or drug combination) that has been found effective in open studies	Panic disorder	Combined SSRI and TCA, olanzapine monotherapy, combined SSRI and olanzapine or a TCA, addition of pindolol to an SSRI, combined valproate and clonazepam In refractory cases, open studies have documented the efficacy of olanzapine and of the addition of fluoxetine to a TCA, of a TCA to fluoxetine, and of olanzapine to an SSRI
	GAD	Ziprasidone
	Social phobia	Tranylcypromine; in refractory cases, addition of buspirone to an SSRI

Medicolegal issues should be considered whenever drugs that have not been approved for the treatment of a certain anxiety disorder are given off label

#### Combining psychotherapy and medication

The guideline does not recommend to start with psychotherapy before considering pharmacotherapy or vice versa, as there is no evidence from clinical studies justifying such a stepwise approach. As more data favor the combination of both modalities than not, both can be started at the same time. If response to psychotherapy or pharmacotherapy is insufficient, treatment should be switched to the other modality. One meta-analysis found higher effect sizes for medications than for psychological therapies and no evidence that gains with psychotherapy can be weakened by concomitant drug treatment [9].

#### Treatment of anxiety disorders in older patients

Anxiety disorders are most common in the age between 30 and 50. The average age in clinical studies was 37 years for PDA, 41 for GAD and 35 for SAD [10]. Therefore, anxiety disorders are less common in patients over 65 years, with the exception of GAD, which may be common in the elderly (Table 6).

**Table 6:**
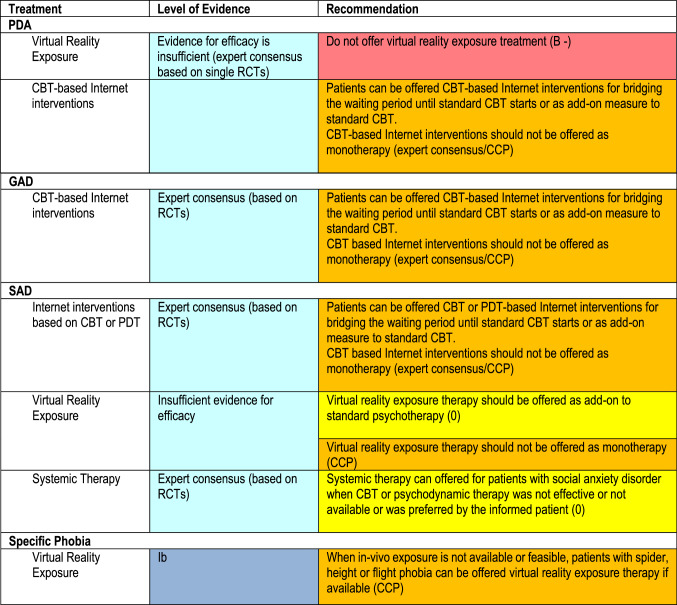
New recommendations in version 2 of the German Guideline for anxiety disorders

Only few studies for the treatment of GAD have been performed with older patients. Some studies in GAD suggest that CBT is less effective than in adults aged 18–65. Studies showed that duloxetine, venlafaxine, pregabalin, and quetiapine are effective in patients over 65 years. In older patients, an increased sensitivity to drug side effects and interactions has to be considered, including anticholinergic effects, risk of orthostatic hypotension and electrocardiogram changes, risk of falling, and paradoxical reactions to benzodiazepines.

#### Pregnancy and breastfeeding

When pregnant women suffer from an anxiety disorder, the risk of an untreated anxiety disorder must be weighed against the risk of damage to the unborn child as a result of drug treatment. A large study suggested no substantial increase in the risk of cardiac malformations attributable to use of antidepressants during the first 3 months of pregnancy [17]. Antidepressants have been associated with increased risk of spontaneous abortions, stillbirths, early deliveries, respiratory distress, and endocrine and metabolic dysfunctions [27]. However, the current evidence suggests that the use of many antidepressants, especially the SSRIs, is favorable compared to exposing the mother to the risks of untreated depression or anxiety disorders [25, 28].

Likewise, a careful assessment of the risk/benefit balance has to be done when a mother is breastfeeding. In such cases, CBT should be considered as an alternative to medication treatment.

### Other treatment options

Exercise (running 5 km three times a week) was shown to be effective in treating PDA [13, 31]. However, exercise was less effective than clomipramine and no more effective than a control condition, relaxation. Therefore, exercise can only be recommended as add-on treatment to standard treatments. For GAD, the only available study could not reliably demonstrate superiority of weightlifting or cycling of a waitlist control condition. According to a meta-analysis of RCTs with patients with anxiety and related disorders such as OCD or PTSD [29], exercise had a small but statistically significant effect compared to control conditions.

Although controlled studies on the usefulness of self-help groups are lacking, it was an expert consensus that patients should be encouraged to participate in such activities if appropriate. It may also be useful to integrate the family members of the affected patients into the treatment plan.

Various other treatments that have been studied in RCTs. However, the guideline committee did not find sufficient evidence to recommend the following treatments: Client-Centered Therapy, Interpersonal Therapy, Progressive Muscle Relaxation, Applied Relaxation, Eye Movement Desensitization and Reprocessing, Music/Dance/Art Therapy, Yoga, beta-blockers, phytotherapeutics, homeopathic formulations, and repetitive magnetic stimulation (for references, see [4]).

 New recommendations in version 2 of the guideline are summarized in Table 6.

## Discussion

Since the publication of the first version of the German guideline for the treatment of anxiety disorders for adults [7], no fundamental changes of the treatment recommendations have been formulated. Treatment with SSRIs/SNRIs and CBT is still the mainstay for anxiety disorders.

Among newer developments, the number of clinical studies examining Internet psychological interventions (IPIs) for anxiety disorders has surpassed the number of studies on psychotherapy with face-to-face contact in the recent years, perhaps because such trials are much easier to conduct and less expensive than studies with face-to-face psychotherapy. At present, these treatment programs have some advantages, because personal contacts can be avoided during the COVID-19 pandemic. Moreover, IPIs are less expensive, save therapist time, require less organizational efforts, save travel time, and can be used at any time of the day. However, a closer look at the quality of the studies is warranted [11]. Even if the IPI programs are very sophisticated and can be individualized for special anxiety manifestations of each participant, it is hard to believe that computer programs can adequately address the unique interpersonal, social, medical or occupational problems of the participating individuals at the same level as ‘real’ therapists.

In most IPI studies, participants could shortly contact study staff by E-mail or telephone. However, in many of the published trials, the ‘therapists’ at the other end were psychology students with incomplete psychotherapy education. In some studies, diagnoses were only made using online diagnostic tests, or by psychology students using structured interviews [11]. Only in 15% of the studies, diagnoses were made by psychiatrists or psychologists in a personal interview. In almost all studies, the efficacy results were based on unblinded self-ratings. On average, two thirds of the participants in IPI studies had an academic background and recruitment has mostly relied on advertisements in the internet or other media, which may affect the generalizability of the study results. Altogether, the effect sizes of IPIs obtained in these studies may have been overestimated.

Since the first version of the guideline, more studies have been published with the new technologies Virtual Reality (VR) and Augmented Reality (AR) which are used in the treatment of specific phobias such as the fear of spiders, heights, or flying. As there is no sufficient evidence yet that these methods are as effective as in vivo exposure therapy, the expert panel recommends to use only these technologies as adjunctive treatment to standard behavior therapy or in cases in which alternative treatments are not available. However, as research in this field is growing constantly [14] and the programs increasingly provide a realistic, true to life experience, especially with the introduction of AR, these methods may play an important role in the treatment of phobias in the future.

Since the 2014 version of the guideline, no new medications for anxiety disorders have emerged. Therefore, there was no change in the recommendations for psychopharmacological treatment. Although there are many unmet needs in the pharmacologic treatment, no putative novel anxiolytic agents will be available in the near future [34]. One reason why pharmaceutical companies are increasingly reluctant to develop new drugs is that the patents of all current available anti-anxiety drugs have expired and very inexpensive generics are produced in China or India because of the lower production costs, while, at the same time, the costs of developing new drugs have increased dramatically.

The applicability of the present guideline is not only restricted to the special situation in Germany. It may also be useful for developing evidence-based treatment plans for adults with anxiety disorders in other countries, as it is based on a thorough world-wide evaluation of RCTs. Today, most drugs are developed for the international market, and the major principles of psychotherapy are not substantially different in the world-wide perspective.

## Abbreviations

AR: Augmented reality exposure therapy
CBT: Cognitive-behavioural therapy
DSM: Diagnostic and statistical manual for mental disorders
GAD: Generalized anxiety disorder
ITT: Intention to treat
PDA: Panic disorder with or without Agoraphobia
PDT: Psychodynamic therapy
RCT: Randomized controlled trial
RIMA: Reversible inhibitor of monoamine oxidase A
SAD: Social anxiety disorder
SNRI: Serotonin-noradrenaline reuptake inhibitor
SSRI: Selective serotonin reuptake inhibitor
VR: Virtual reality exposure therapy
